# Physical decline and survival in the elderly are affected by the genetic variability of amino acid transporter genes

**DOI:** 10.18632/aging.101420

**Published:** 2018-04-18

**Authors:** Paolina Crocco, Eneida Hoxha, Serena Dato, Francesco De Rango, Alberto Montesanto, Giuseppina Rose, Giuseppe Passarino

**Affiliations:** 1 Department of Biology, Ecology and Earth Sciences, University of Calabria, 87036 Rende, Italy; * Equal contribution

**Keywords:** amino acid transporter genes, mTORC1, aging, muscle decline, sarcopenia, Hand grip, ADL

## Abstract

Amino acid (AA) availability is a rate-limiting factor in the regulation of muscle protein metabolism and, consequently, a risk factor for age-related decline in muscle performance. AA transporters are emerging as sensors of AA availability and activators of mTORC1 signalling, acting as transceptors. Here, we evaluated the association of 58 single nucleotide polymorphisms (SNPs) in 10 selected AA transporter genes with parameters of physical performance (Hand Grip, Activity of Daily Living, Walking time). By analysing a sample of 475 subjects aged 50-89 years, we found significant associations with *SLC7A5*/LAT1, *SLC7A8*/LAT2, *SLC36A1*/PAT1, *SLC38A2*/SNAT2, *SLC3A2*/CD98, *SLC38A7*/SNAT7 genes. Further investigation of the SNPs in a cross-sectional study including 290 subjects aged 90-107 years revealed associations of *SLC3A2*/CD98, *SLC38A2*/SNAT2, *SLC38A3*/SNAT3, *SLC38A9*/SNAT9 variability with longevity. Finally, a longitudinal study examining the survival rate over 10 years showed age-dependent complexity due to possible antagonistic pleiotropic effects for a SNP in *SLC38A9*/SNAT9, conferring a survival advantage before 90 years of age and a disadvantage later, probably due to the remodelling of AA metabolism. On the whole, our findings support the hypothesis that AA transporters may impact on the age-related physical decline and survival at old age in a complex way, likely through a mechanism involving mTORC1 signalling.

## Introduction

One of the most dramatic modifications associated with human aging is the progressive decline in skeletal muscle mass and function, known as sarcopenia. Sarcopenia causes physical function impairment, leading to a loss of functional independence and to an increased incidence of adverse health outcomes [1,2]. The prevalence of this condition, which is progressively increasing due to the prolonged life expectancy, has led in the recent decades to increased needs for health care services and resources to support older people [3]. The rate of skeletal muscle loss is estimated at 8% per decade from the 4th until 7th decade, with about 15% lost each decade after 70 years of age and loss of strength is estimated to be even longer [4].

The cause of sarcopenia is widely regarded as multifactorial, with chronic diseases, hormonal and inflammatory changes, mitochondrial dysfunction, physical inactivity, and malnutrition, being the main risk factors [5]. In addition to these causes, loss of muscle has been related to the imbalance between muscle protein synthesis (MPS) and muscle protein breakdown (MPB), associated with alterations in muscle anabolic responses to nutritional stimuli and physical activity, the so-called “anabolic resistance” according to which in the elderly process the “anabolic threshold” required to maximize anabolic pathways is increased [6,7].

Amino acids (AAs) availability is among the most important anabolic signals for MPS [8], and essential amino acids (EAAs), leucine in particular, has been shown to be critical in the regulation of skeletal muscle protein synthesis and degradation in the elderly [9]. There is growing evidence that AA transport into muscle cells may be a rate-limiting step in the process of AA induced stimulation of skeletal muscle protein metabolism, and that AA transporters may have a key role in such process as AA sensors [10]. This is likely linked to the ability of AA transporters to act as both transporter and receptor (transceptor) [11,12] able to transduce a signal reflecting AA availability and leading to activation of the mammalian/mechanistic target of rapamycin complex 1 (mTORC1), the major nutrient-sensitive signalling pathway [13]. Thus, AA transporters may be an important link in the ability for AAs to stimulate MPS. This has been supported by studies in human skeletal muscle showing that the expression of AA transporters is highly dynamic and responsive to different anabolic stimuli [14]. In particular, in 2010, Drummond and colleagues [15] characterized the expression of selected AA transporters in the human skeletal muscle following AA ingestion in a group of healthy young individuals. They found increased mRNA expression of L-type AA transporter *SLC7A5* (LAT1), *SLC3A2* (CD98), sodium-coupled neutral AA transporter *SLC38A2* (SNAT2), and proton-coupled amino acid transporter *SLC36A1* (PAT1), which are transporters thought to have key roles in mTORC1 signalling regulation and muscle protein synthesis and muscle growth. These authors argue that, changes in the expression levels of AA transporters, possibly mediated by mTORC1 activity, could serve as an adaptive response for improving AA intracellular delivery and for regulating the rate of muscle protein synthesis in response to anabolic stimuli or during periods of decreased muscle protein synthesis [15]. Consistently with this hypothesis, the same authors showed that an up-regulation of the above transporters occurred after resistance exercise in both young and older adults, likely regulated in an age-dependent manner [16], as well as in older persons in conditions of short-term bed rest [17]. Soon after that, Dickinson and co-workers [14] reported that aging differentially affected the expression of *SLC7A5* and *SLC38A2* in the skeletal muscle when EAAs were ingested after exercise, suggesting that aging may influence the function of specific AA transporters, and possibly age-related phenotypes, such as sarcopenia [14,18].

Based on these evidences, to shed a light on the impact of the above AA transporters on the age-related loss of muscle strength and physical performance, we screened genetic variants occurring in their genes in a population of elderly subjects (< 90 years), analysing them in combination with parameters of physical status. Moreover, beside these four genes [*SLC7A5* (LAT1), *SLC3A2* (CD98), *SLC38A2* (SNAT2) and *SLC36A1* (PAT1)], we also included variants located in *SLC7A8* (LAT2) and *SLC43A1* (LAT3) as leucine transporters like LAT1, *SLC1A5* (ASCT2) as a glutamine transporter like SNAT2, *SLC38A3* (SNAT3) and *SLC38A7* (SNAT7), preferentially transporting glutamine and arginine, *SLC38A9* (SNAT9), as an arginine transporter [19,20].

To investigate whether variants in the selected genes also affect the chance to survive and/or reach very advanced ages, we exploited both the cross-sectional and longitudinal approach by taking advantage of the available dataset, which also included very old subjects (≥90 years), and survival data within 10-year follow-up period from the baseline visit.

## RESULTS

Demographic, clinical, and anthropometric characteristics of the analysed sample are presented in Table 1. Among the selected SNPs, 10 did not pass the QC phase. In particular, four SNPs were excluded due to a MAF (Minor Allele Frequency) lower than ten percent per locus (rs17112008, rs7968173, rs1175, rs7735053), while six were excluded because they showed a significant deviation from HWE in control subjects (rs11749532, rs7736177, rs2897968, rs17794251, rs7193392, rs8058969).

**Table 1:** Socio-demographic characteristics and functional parameters in the sample stratified for group membership.

	50-89 (N = 475)	90-108 (N = 290)
Age (year)		
Mean (SD)	70.40 ± 8.71	96.86 ± 3.86
Male (%)	50%	37.24%
		
Height		
Mean (SD)	161.54 ± 9.28	151.39 ± 9.52
Range	138-190	125-175
		
BMI (SD)		
Mean (SD)	27.24 ± 4.19	23.12 ± 4.03
Range	17.80-45.35	12.98-40.54
		
HG strength		
Mean (SD)	22.86 (10.0)	13.09 (6.22)
Range	4-55	1-36
		
ADL [n (%)]		
Non disable (=5)	84.4%	31.9%
Disable (<5)	15.6%	68.1%
		
Walking 4 meters [sec]		
Mean (SD)	7.77 (4.35)	12.3 (7.26)
Range	2.65-50.00	3.65-43.0

Abbreviations: BMI: Body mass index; ADL, Activity Daily Living; HG, Hand Grip Strength.

### Association with muscle-related phenotypes

Complete results of the association tests in the 50-89 years old cohort are reported as Supplementary Figure 1 (Figure S1). In Table 2 the SNPs showing association with at least one trait under study at nominal statistical significance (p ^Model^< 0.05) are reported.

**Table 2:** SNPs showing at least one significant association with functional parameters under a nominal level (p Model < 0.05).

Gene	SNP	MAF	Hand Grip*		ADL°		Walking Time°	
			β ± se	pModel	OR (95% CI)	pModel	β ± se	pModel
*SLC3A2* CD98	rs12804553	T=0.25					+2.48 ± 1.15	0.029^R^
	rs4726	T=0.20					-1.40 ± 0.68	0.034^D^
*SLC7A5* LAT1	rs4329925	C=0.15	-1.98 ± 0.96	0.027^D^				
	rs731710	G=0.48			0.47 (0.27-0.81)	0.005^A^		
*SLC7A8* LAT2	rs999165	A=0.19	-3.09 ± 0.95	0.0013^D^				
	rs12588118	G=0.27	-6.41 ± 2.06	0.002^R^				
	rs3783436	C=0.34			0.40 (0.20-0.82)	0.007^A^		
*SLC36A1* PAT1	rs357618	G=0.30			0.11 (0.01-0.96)	0.013^R^		
	rs357629	G=0.32			0.11 (0.01-0.96)	0.013^R^		
*SLC38A2* SNAT2	rs1873793	C=0.48			3.06 (1.36-6.85)	0.007^R^		
*SLC38A7* SNAT7	rs9806843	G=0.34					+ 1.70 ± 0.63	0.008^D^

Abbreviations: MAF, Minor allele frequency; β, beta coefficient, se, standard error; OR, odds ratio; CI, confidence interval. p Model is the p value of the best genetic model, where R is recessive, D is dominant, and A is additive model.

*Age, sex, and height were included as covariates.

°Age and sex were included as covariates.

Association with HG performance was found for LAT genes *SLC7A5* rs4329925 T/C (β = -1.98, p^Dom^ = 0.027), and for *SLC7A8* rs999165 T/A (β = -3.091, p^Dom^ = 0.0013) and rs12588118 C/G (β = -6.41, p^Rec^ = 0.002). For these SNPs, the minor allele was associated with a decreased HG performance as indicated by the corresponding regression coefficients.

*SLC7A5* and *SLC7A8* variability was also associated with ADL performance. For *SLC7A5*, we found association with rs731710 A/G, while for *SLC7A8* the variant most significantly associated was rs3783436 T/C. For both SNPs, the minor allele was conferring an increased ability to perform physical activities with an OR of 0.47 (CI 0.27-0.81, p^Add^ = 0.005) and 0.40 (CI 0.20-0.82, p^Add^ = 0.007) per risk allele, respectively. A positive effect on ADL performance was also observed for two SNPs in *SLC36A1*, rs357618 A/G and rs357629 A/G. For both, the estimated OR was 0.11 (CI 0.01-0.96; p^Rec^ = 0.013). LD analysis indicated these two SNPs to be strongly correlated with each other (r^2^ = 0.97). Therefore, these associations are not independent.

As to *SNAT* genes, a strong association with ADL scores was found for *SLC38A2* rs1873793 T/C, with CC homozygous subjects having a higher risk to become disable (OR 3.06, CI 1.36-6.85; p^Rec^ =0.007).

In the case of WT performance, two SNPs in *SLC3A2*, rs12804553 G/T and rs4726 C/T, showed an opposite effect: subjects with two copies of the less frequent allele were associated, respectively, with longer (β = +2.48; p^Rec^ = 0.029) and shorter (β = -1.40; p^Dom^ = 0.034) walking time. Another positive significant association was found for *SLC38A7* rs9806843 (β = +1.70; p^Dom^ = 0.008).

### Association with longevity

To investigate whether variants in genes encoding AA transporters also concur to determine the chance to reach very advanced age we applied a cross-sectional study, by including 271 more subjects aged 90 years and older.

Complete results of case-control analysis are reported in Figure S1 (D), while Table 3 shows the statistically significant associations. Among the SNPs showing association with at least one muscle-related phenotype, in accordance with the negative effect of the minor C allele of rs1873793 (*SLC38A2*) on ADL performance, subjects carrying this allele were also significantly less frequent in the older population compared to the younger one (OR=0.70, CI 0.5-0.95; p=0.035). In addition to this, we found that the minor allele of rs1858828 in *SLC38A3* (OR=1.33 (1.06-1.68), p^Add^=0.01) was positively correlated with longevity. A lower chance to reach very advanced ages was found for the minor allele of rs12794763 in *SLC3A2* (OR=0.42, 0.26-0.68; p^D^=0.0002), and for the minor alleles of three SNPs in *SLC38A9*: two of them, rs4865615 and rs7704138, are in LD with each other (r^2^>0.8) and showed respectively OR=0.54 [(0.33-0.88), p^Rec^=0.011] and OR=0.56 [(0.34-0.92), p^Rec^=0.018], while rs10056358 showed a OR=0.66 [(0.45-0.96), p^Dom^=0.029)].

**Table 3:** SNPs showing significant associations with longevity under a nominal level (p Model < 0.05).

Gene	SNP	MAF	OR (CI)	pModel
*SLC3A2* CD98	rs12794763	G=0.15	0.42 (0.26-0.68)	0.0002^D^
*SLC38A2* SNAT2	rs1873793	C=0.48	0.70 (0.50-0.98)	0.035^D^
*SLC38A3* SNAT3	rs1858828	T=0.38	1.33 (1.06-1.68)	0.014 ^A^
*SLC38A9* SNAT9	rs4865615	C=0.4	0.54 (0.33-0.88)	0.011^R^
	rs7704138	C=0.39	0.56 (0.34-0.92)	0.018^R^
	rs10056358	A=0.14	0.66 (0.45-0.96)	0.029 ^D^

Abbreviations: MAF, Minor allele frequency; OR, odds ratio; CI, confidence interval. p Model is the p value of the best genetic model, where R is recessive, D is dominant, and A is additive model.

The variable sex was considered a covariate.

### Association with survival

**Figure 1:**
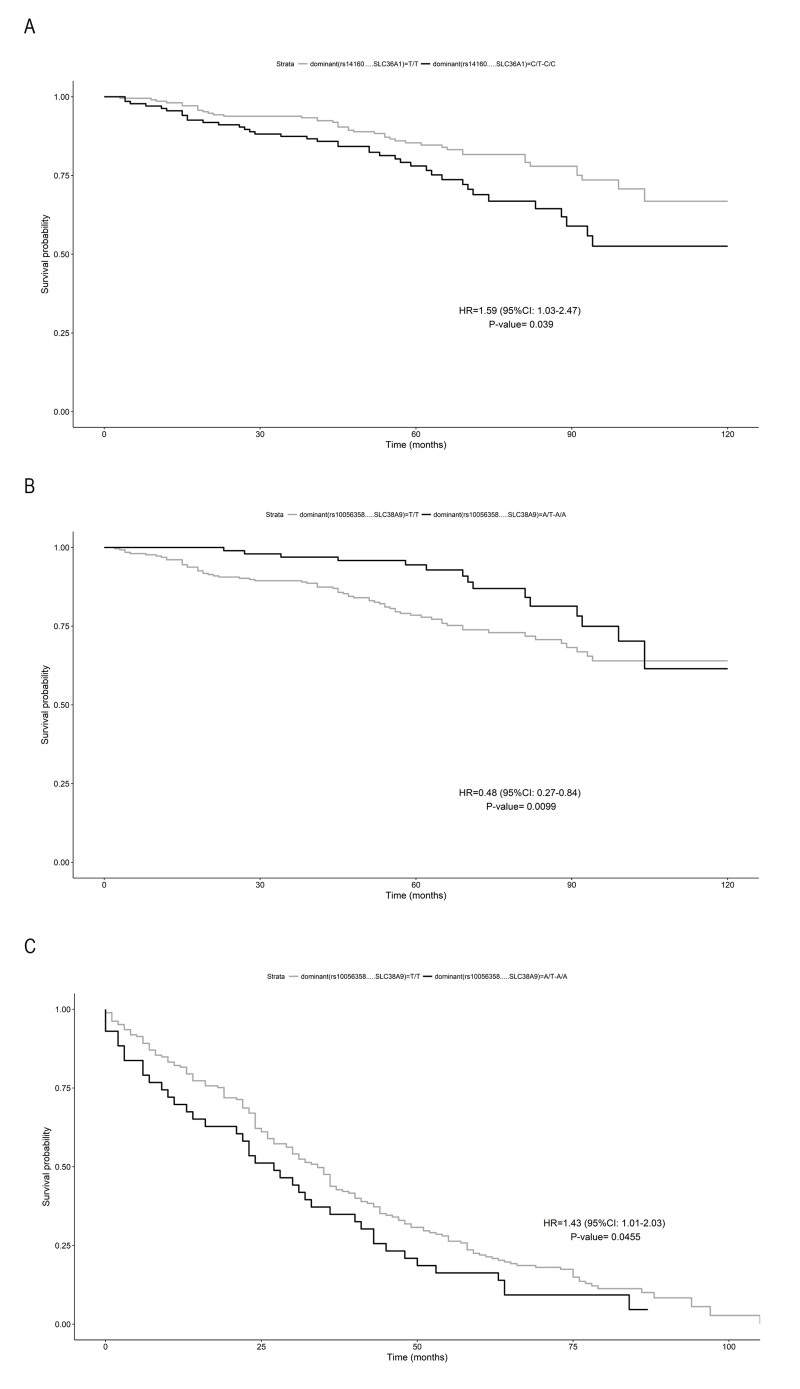
**Survival function of carriers of minor allele (black) vs non carriers (grey).** (**A**) rs14160 (SLC36A1) in subjects aged <90 years; (**B**) rs10056358 (SLC38A9) in subjects aged ≥90 years; (**C**) rs10056358 (SLC38A9) in subjects aged > 90 years. Time is expressed in months, where 0 is considered the time of recruitment, and each individual is followed up for survival status till death. HR value, confidence interval and p-value from Cox regression analysis are reported inside the figure.

Next, using 10-year of follow-up survival data, we investigated the association with survival of the analysed SNPs, estimating their quantitative effects. An increased risk of death was found for subjects carrying the C allele at rs14160 (*SLC36A1)* compared to those with the TT genotype (adjusted HR = 1.59, 95% CI: 1.025–2.47; p=0.038) (Figure 1a). In *SLC38A9*, the presence of the A allele for the rs10056358 variation confers a survival advantage before 90 years of age, compared to those with the TT genotype (adjusted HR=0.48, 95% CI 0.27-0.84, p=0.009) (Figure 1b), and a disadvantageous effect later, (adjusted HR = 1.43, 95% CI 1.01-2.03; p= 0.045) (Figure 1c).

Analysis of all other SNPs showed no statistically significant or suggestive differences in survival between genotypes.

### Imputation of functional impact of SNPs

Finally, to explore the functional consequences of the associated polymorphisms we used regulatory information from ENCODE data using HaploReg v4.1 and RegulomeDB databases. Results from the functional annotation analysis are shown in Table S2. By using HaploReg v4.1 database, we found promising functional implications for these SNPs (i.e. promoter and enhancer histone marks, transcription factor binding sites, and eQTL hits), indicating a regulatory potential. The functional implication of some SNPs was further corroborated by RegulomeDB results showing for rs357629 (*SLC36A1*) a "likely to affect binding and linked to expression of a gene target (score 1f)" and for rs4329925 and rs731710 (*SLC7A5*), and for rs1873793 (*SLC38A2*) a "likely to affect binding (score 2b)". LD patterns showed that all SNPs, except two (rs12794763, *SLC3A2* and rs999165, *SLC7A8*), were in LD with several variants that can collectively capture the casual variant.

## DISCUSSION

The goal of the current study was to investigate the impact of SNPs in selected AA transporters genes on physical performance and survival at old age.

Our analysis of a group of elderly individuals in the age ranges of 50-89 years, showed that genetic variants in *SLC7A5 (*rs4329925) and *SLC7A8* (rs999165 and rs12588118), negatively affected HG strength, a reliable marker of the muscle performance and an indicator of quality of aging [21]. Additionally, different SNPs in the same genes (rs731710 in *SLC7A5* and rs3783436 in *SLC7A8*) were significantly associated with ADL levels. *SLC7A5* and *SLC7A8* code, respectively, for the plasma membrane proteins LAT1 and LAT2. Each of them forms with CD98 (*SLC3A2*) a heterodimeric bidirectional antiporter that regulates the simultaneous transport of leucine into cells and efflux of glutamine out of cells. Intriguingly, we found that two variants in *SLC3A2* (rs12804553 and rs4726) were associated, with opposite effects, with walking ability. These findings point to a role of the LAT1(LAT2)/CD98 complex in affecting muscle strength and physical performance in the elderly, likely through regulation of the anabolic signal mediated by the intracellular leucine/glutamine levels, and downstream activation of mTORC1. On the other hand, it is well known that leucine provides direct anabolic stimuli to skeletal muscle, so that the post-exercise leucine assumption is considered a potential treatment in numerous muscle wasting conditions [18]. Similarly, glutamine status is considered a hallmark of catabolic states and muscle loss [22].

Further supportive evidence to the above hypothesis is provided by the association with genes that mediate the transport of glutamine, such as *SLC38A2* (SNAT2) and *SLC38A7* (SNAT7). We found, indeed, significantly worse ADL scores associated to rs1873793 (*SLC38A2*), a finding supported by a disadvantage in attaining longevity for subjects carrying the same allelic variant, and a better walking performance associated to rs9806843 (*SLC38A7*). For the above transporters an important role in mTORC1 activation was documented. For instance, a reduction of SNAT2 activity in muscle cells is associated with reduced levels of glutamine and leucine and impaired protein synthesis through mTORC1 [23]; furthermore, it was found that the expression of system SNAT2, LAT1 and CD98 was upregulated after leucine availability in L6 myotubes [24]. The importance of these AA transporters is further bolstered by the fact that the heterodimer LAT1-CD98 also regulates the leucine flux into the lysosome [25], the major cellular compartment for mTORC1 activation [26], and that SNAT2 may act as a *transceptor* involved in both amino acid transport and signal transduction [27].

Although the mechanisms by which aging alters leucine-induced protein synthesis are currently under investigation, our data are in accordance with the hypothesis that a concerted activation of AA transporters may occur differently in young and older adults, finally influencing the anabolic response of skeletal muscle to AA availability. This hypothesis is supported by Drummond and Dickinson’s work showing that aging differentially affects the expression of *SLC7A5* and *SLC38A2* in the skeletal muscle, in response to resistance exercise and essential AA supplementation [14,17]. These evidences are also in keeping with our findings regarding *SLC36A1* (PAT1), which has been reported to function as transceptor on the lysosomal membrane [28]. For this gene, we found the minor alleles at rs357618 and rs357629 (in LD with each other) associated with a better ADL performance. A different variant of *SLC36A1* (minor allele of rs14160), was found, instead, to increase the risk of death along a 10 year follow up of survival in our younger sample. Similarly, different *SLC3A2* variants showed cross-phenotype associations on different age-related phenotypes [29]. As to SNAT9 (*SLC38A9*), another lysosomal transceptor [30–32], we found a complex and dynamic association with survival chance. In fact, while a survival advantage was found for carriers of the rs10056358 A allele in the sample group aged 50-89 years, 90-plus subjects carrying this allele showed a lower probability to survive. Accordingly, the same minor allele A negatively influenced the probability to achieve longevity. Thus, this SNP manifests an antagonistic pleiotropic effect on survival at old ages, with the rs10056358-A allele conferring a positive effect on survival at ages before 90 and negative effect afterwards. The absence of correlation between *SLC38A9* gene and the parameters of physical performance suggests that it functions as a pleiotropic gene with age-dependent effects, affecting survival and longevity but not quality of aging [33,34]. On the contrary, conditional effects of these genes on quality of aging but not on longevity are shown for the most part of the genes studied in this work.

Overall, our data provide evidence that the genetic variability of these genes may impact on muscle performance and/or physical decline in adulthood, as well as on the probability to survive at old age. Thus, taking into account that AA transporters may act as sensors of AA availability and that this availability is closely related to skeletal muscle metabolism, AA transporter genetic variability may act at forefront of individual susceptibility to anabolic resistance experienced with age, acting as risk factor for the onset of muscular decline in the elderly population.

Hence, understanding the functional implications of the associated SNPs may represent an important indication for elucidating the possible molecular mechanism underlying the associations found. However, the majority of the associated SNPs are non-coding variants, except two SNPs located in the coding regions. Our bioinformatic analyses identified, for some of them, a number of features (histone modifications, DNase I hypersensitivity clusters and transcription factor binding sites) consistent with a possible regulatory function, although we cannot exclude the possibility that the associated variants represent proxies for unknown causal variants as a result of LD, apart from those not showing any LD in their genomic region (rs12794763 and rs999165).

We are aware that our study has some weaknesses that should be addressed. A first limitation of the study is the lack of proper correction for multiple testing. Since this study was exploratory, a Bonferroni correction would have eliminated potentially important findings if applied. Another possible limitation could be the time of the survival follow up, not sufficient to draw long-term conclusions on the effect of genetic variants with minor effect on survival. Furthermore, the sample size could be increased and further explorations in additional study populations are needed before conclusions can be drawn.

Notwithstanding, and considering that this is the first study reporting genetic variants in AA transporter genes associated to the age-associated decline of muscle performance, we believe that our findings can open future investigations on the role of AA transporters in the quality of aging and longevity. This could provide valuable insights into potential targets for risk stratification in the population, and for therapeutic interventions aimed at increasing muscle mass and strength at old age.

## METHODS

### Study population

The study was conducted on a sample of 765 subjects in the age range 50–107 years. The younger sample (age-range 50-89 years; mean age 70.40 ± 8.71) included 475 subjects (238 males and 237 females), the oldest old one (age range 90-108 years; mean age 96.86 ± 3.86) included 290 subjects (108 males and 182 females). See Table 1 for a complete description of the sample studied. All the subjects were born in Calabria (southern Italy) and their ancestry in the region had been ascertained up to the grandparents’ generation. Samples were collected within the framework of several recruitment campaigns carried out for monitoring the quality of aging in the whole Calabria region from 2002 onwards. Subjects older than 90 years were identified through the population registers and then contacted by specialised personnel and invited to join the study. Younger subjects were contacted through general physicians. Finally, each subject was recruited after a complete multidimensional geriatric assessment with detailed clinical history, including anthropometric measures and a set of the most common tests to assess cognitive functioning, functional activity, physical performance and depression. In addition, common clinical haematological tests were performed. White blood cells (WBC) from blood buffy coats were used as source of DNA.

### Ethic statement

Recruitment campaigns and subsequent analyses received the approval of the relevant ethical committee. All the subjects provided written informed consent for the permission to collect blood samples and usage of register-based information for research purposes.

### Physical performance

Hand Grip (HG) strength was measured by a handheld dynamometer (SMEDLEY's dynamometer TTM) while the subject was sitting with the arm close to his/her body. The test was repeated three times with the stronger hand and the maximum of these values was considered in the analyses. When a test was not carried out, it was specified if it was due to physical disabilities or because the subject refused to participate. Since HG strength is affected by age, sex, and height, the scores were corrected for these factors.

Walking time was measured as the best performance (shortest time in seconds) of two walks along a 4-meter distance.

### Functional activity

The management of Activities of Daily Living (bathing, dressing, toileting, transfer from bed to chair, and feeding) was assessed using a modification of the Katz Index of ADL [35]. The assessment was based on what the subject was able to do at the time of the visit. The score is given counting the number of activities in which the participant is dependent or independent at the time of the visit. For the analyses, ADL scores were dichotomized as one if the subject was not independent in all five items and zero otherwise.

### SNP selection and genotyping

A total of 58 SNPs mapping within and nearby genes encoding 10AA transporters were prioritized by a tagging approach, attempting to choose those most likely to be of functional relevance (nonsynonymous SNPs, SNPs located in the 5′ and 3′ UTR regions). Supplementary Table S1 reports the complete list of selected SNPs, their position (relative to the chromosome and to the gene), and putative functional annotation.

Multiplex SNP genotyping was performed using PCR followed by primer extension and MALDI-TOF mass spectrometry using iPLEX Gold technology from Sequenom (Sequenom Inc, San Diego, USA). Sequenom MassARRAY Assay Designer software (version 3) was used to design primers for PCR and single base extension. Standard procedures were used to amplify PCR products, and unincorporated nucleotides were deactivated with the shrimp alkaline phosphatase (SAP). A primer extension reaction was implemented by mass extension primer and terminator. The primer extension products were then desalted on resin, and spotted onto the 384-element SpectroCHIP (Sequenom) for MALDI-TOF analysis using Spec-troACQUIRE v3.3.1.3 (Sequenom). Spectra were analyzed using MassARRAY Typer v3.4 Software (Sequenom). Approximately 10% of the samples were analysed in duplicate, and the concordance rate of the genotypes was higher than 99%.

### Quality-control

After genotype calling, the dataset went through a battery of quality-control (QC) tests. At sample level, subjects with a proportion of missing genotypes higher than 10% were excluded from the study. At SNP level, SNPs were excluded if they had a significant deviation from Hardy-Weinberg equilibrium (HWE, p<0.05), a Missing Frequency (MiF) higher than 10% and a Minor Allele Frequency (MAF) lower than 5%.

### Statistical analyses

Continuous and categorical variables were compared by using the independent samples t-test and the chi-square test as appropriate. For each SNP, allele and genotype frequencies were estimated by gene counting from the observed genotypes. Hardy Weinberg Equilibrium (HWE) was tested by Fisher’s exact test. Pairwise measures of linkage disequilibrium (LD) between the analysed loci was estimated by Haploview (https://www.broadinstitute.org/haploview/haploview). Linear and logistic regression models were applied to estimate the impact of genetic variability on parameters of muscle strength (HG) and physical performance (WS and ADL), including as covariates age, gender, and height in the formulated regression models. A logistic regression model was also used to evaluate the effect of genetic variability on the chance to reach very advanced age. In these models, genetic data were coded with respect to a dominant, recessive, and additive fashion. Then, for each SNP the most likely genetic model was estimated on the basis of minimum level of statistical significance (Wald’ test p-value).

The hypothesis tested in this study is based on prior evidences for a role of AA transporters in the mTORC1-mediated induction of muscle protein synthesis. Furthermore, the involvement of genetic variability of AA transporters in relation to age-related muscle loss are lacking: in this sense, this study was exploratory. Thus, the p-values of single SNP analysis are reported without Bonferroni post hoc correction for multiple comparisons, to be not conservative and eliminate potentially important findings if applied.

In order to evaluate if the detected effects of the analysed polymorphisms on both muscle strength and physical performance might finally result in differential patterns of survival of the different relevant genotypes, we evaluated survival after 10 years from the baseline visit. Kaplan-Meier survival curves were estimated for each SNP affecting the analysed muscle-related phenotypes. In order to evaluate their predictive value with respect to mortality risk, the obtained survival curves were then compared by log-rank test. Subjects alive after the follow-up time were considered as censored, and this time was used as the censoring date in the survival analyses. In addition, Hazard ratios (HR) and 95% Confidence Intervals (95% CI) were estimated by using Cox proportional hazard models taking also into account possible confounder variables (age and gender).

Statistical analyses have been performed using *SNPassoc* and *surv* packages of R (http://www.R-project.org/).

### Bio-informatic analyses

The functionality of theassociated SNPs was explored by retrieving regulatory information from the ENCODE (https://genome.ucsc.edu/ENCODE/) [36] and the Roadmap Epigenome Mapping projects (http://www.ppmroadmap.com/) [37] as implemented in HaploReg (v4.1, www.broadinstitute.org/mammals/haploreg/) [38], and RegulomeDB (www.regulomedb.org/) [39].

## Supplementary Material

Supplementary File
