# Supplementary material for: Physical decline and survival in the elderly are affected by the genetic variability of amino acid transporter genes
Source: Aging (Albany NY). 2018 Apr 18;10(4):658–73. doi: 10.18632/aging.101420 (PMC5940118; doi:10.18632/aging.101420)
Supplement: Supplementary File [file aging-10-101420-s001.pdf]

SUPPLEMENTARY MATERIAL

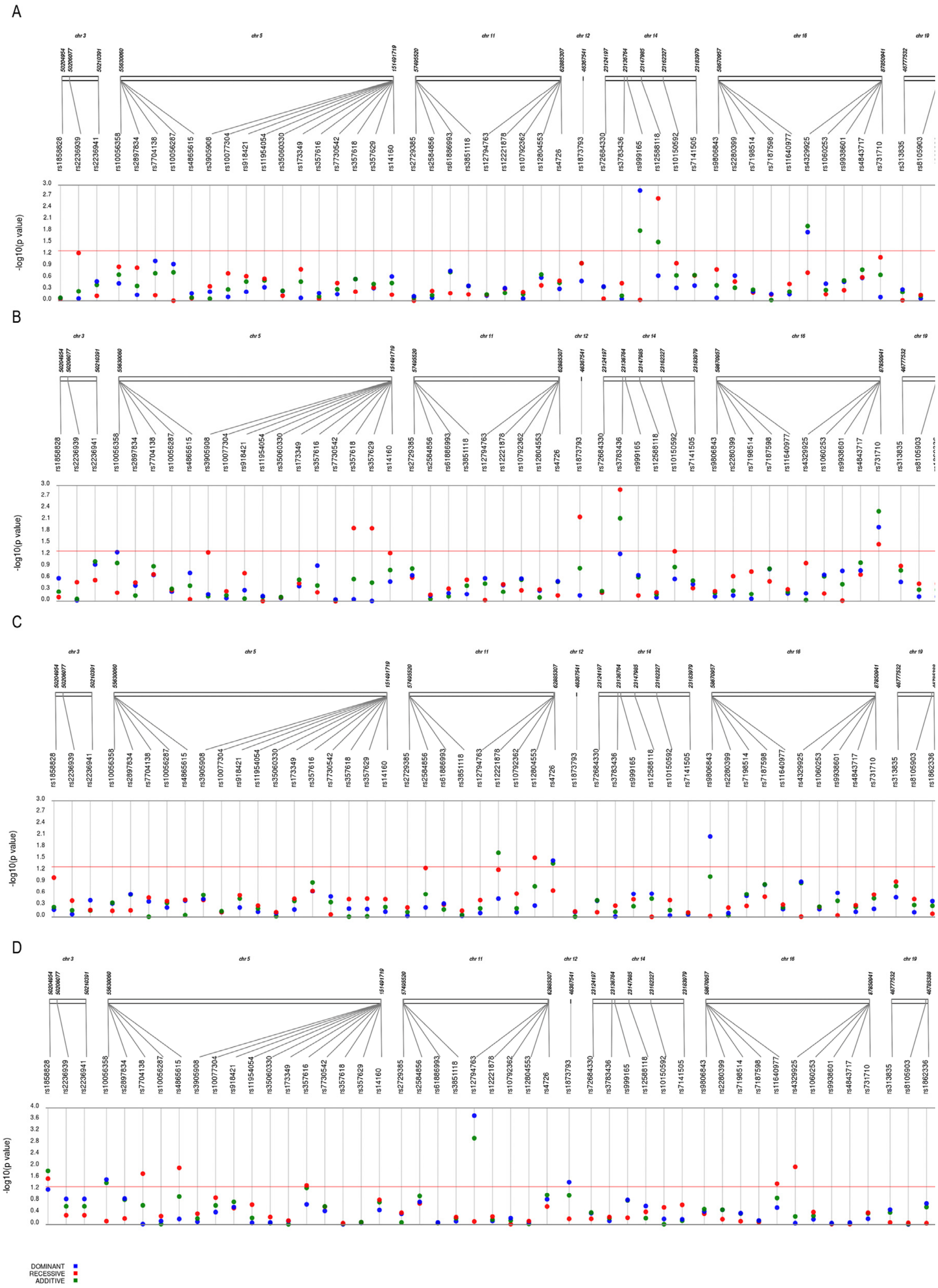

Supplementary Figure S1. Schematic representation of the association results with Hand Grip (**A**) performance, Activity of Daily Living (**B**) scores, Walking Time (**C**) scores, and longevity (**D**).

Supplementary Table 1. Description of the 58 SNPs initially selected.

Gene/ Protein	Chr	SNP	Position (GRCh38.p7)	Mutation type (Ensembl)	Major/Minor allele (Ensembl)	MAF
SLC38A3/ SNAT3	3	rs1858828	50204954	5'near gene variant	G/T	0.44
	3	rs2236939	50206077	Intron variant	G/T	0.11
	3	rs2236941	50210391	Intron variant	C/T	0.32
SLC36A1/ PAT1	5	rs3905908	151445768	5'near gene variant	T/C	0.22
	5	rs10077304	151452320	Intron variant	T/C	0.16
	5	rs918421	151452428	5'UTR variant	A/G	0.46
	5	rs11954054	151456584	Intron variant	A/G	0.23
	5	rs35060330	151458524	Intron variant	C/T	0.33
	5	rs17112008	151458557	Intron variant	C/A	0.33
	5	rs173349	151462020	Intron variant	T/C	0.47
	5	rs357616	151465808	Intron variant	T/C	0.40
	5	rs7730542	151466243	Intron variant	A/G	0.12
	5	rs357618	151467051	Intron variant	A/G	0.39
	5	rs357629	151471385	Intron variant	A/G	0.39
	5	rs14160	151491719	3'UTR variant	T/C	0.28
	5	rs1175	151492234	3'UTR variant	C/T	0.20
SLC38A9/ SNAT9	5	rs10056358	55630060	Intron variant	A/T	0.12
	5	rs2897834	55642751	Intron variant	C/A	0.13
	5	rs7704138	55648434	Intron variant	C/T	0.34
	5	rs10056287	55658650	Intron variant	T/C	0.19
	5	rs4865615	55664845	Missense variant	C/G	0.34
	5	rs7735053	55667626	Intron variant	T/C	0.34
	5	rs11749532	55706437	Intron variant	G/A	0.30
	5	rs7736177	55708116	Intron variant	G/A	0.31
SLC3A2/ CD98	11	rs12794763	62858040	Intron variant	T/G	0.15
	11	rs12221878	62858559	Intron variant	C/G	0.04
	11	rs10792362	62873879	Intron variant	T/C	0.43
	11	rs12804553	62876155	Intron variant	G/T	0.28
	11	rs4726	62885307	Exon Synonymous variant	C/T	0.25
	11	rs2282477	62889032	3'near gene variant	T/C	0.23
SLC43A1/ LAT3	11	rs2729385	57495520	Intron variant	G/A	0.33
	11	rs2584856	57499839	Intron variant	C/A	0.23
	11	rs61886993	57511522	Intron variant	C/G	0.08
	11	rs3851118	57513478	Intron variant	T/C	0.35

SLC7A8/ LAT2	14	rs72684330	23124197	3'near gene variant	T/A	0.12
	14	rs17794251	23124233	3'near gene variant	C/T	0.27
	14	rs3783436	23136764	Intron variant	T/C	0.34
	14	rs999165	23138657	Intron variant	T/A	0.27
	14	rs12588118	23147985	Intron variant	C/G	0.23
	14	rs10150592	23162327	Intron variant	C/A	0.17
	14	rs7141505	23183979	5'near gene variant	C/A	0.29
SLC7A5/ LAT1	16	rs4329925	87828401	3'near gene variant	T/C	0.14
	16	rs1060253	87832532	3'near gene variant	G/C	0.26
	16	rs9938601	87837658	Intron variant	A/G	0.42
	16	rs4843717	87844559	Intron variant	T/C	0.27
	16	rs731710	87850941	Intron variant	A/G	0.41
	16	rs7193392	87854200	Intron variant	G/A	0.39
SLC38A7/ SNAT7	16	rs9806843	58670957	Intron variant	A/G	0.42
	16	rs2280399	58677628	Intron variant	G/T	0.11
	16	rs7198514	58680599	Intron variant	C/T	0.20
	16	rs7187598	58681490	Non coding exon variant	T/C	0.41
	16	rs11640977	58681712	Splice region variant	T/C	0.14
	16	rs8058969	58685026	5'UTR variant	G/A	0.49
SLC1A5/ ASCT2	19	rs313835	46777532	Intron variant	C/T	0.28
	19	rs8105903	46784893	Intron variant	A/C	0.47
	19	rs1862336	46785388	5'UTR variant	T/C	0.24

Abbreviations: MAF (Minor Allele Frequency).
MAF refers to the European population as retrieved by Ensembl (www.ensembl.org)

Table S2. Summary of functional annotation of the phenotype-associated SNPs.

			HaploReg v4.1								RegulomeDB
**GENE**	**SNP**	**SNPs in LD ($r^2 \geq 0.8$)**	**Promoter histone marks**	**Enhancer histone marks**	**DNase**	**Proteins bound**	**Motifs changed**	**GRASP QTL hits**	**Selected eQTL hits**	**dbSNP func annot**	**Score**
SLC3A2 CD98	rs12804553	4	SKIN	6 tissues	SKIN, PLCNT		4 altered motifs		8 hits	intronic	3a less likely to affect binding
	rs4726	8		15 tissues	7 tissues	POL2, POL24H8	Maf		7 hits	synonymous	4 Minimal binding evidence
	rs12794763	none	8 tissues	12 tissues			Foxl1, Pou1f1	1 hits	4hits	intronic	3a less likely to affect binding
SLC7A5 LAT1	rs4329925	31		12 tissues	13 tissues	POL24H8	Ets,RBP-Jkappa		1 hits	1.6kb 3' of SLC7A5	2b likely to affect binding
	rs731710	4		19 tissues	7 tissues		ATF3,ATF6,RFX5		3 hits	intronic	2b likely to affect binding
SLC7A8 LAT2	rs999165	none		8 tissues	GI,BLD		HDAC2,PRDM1,TATA			intronic	No Data
	rs12588118	8		13 tissues	4 tissues		4 altered motifs			intronic	4 Minimal binding evidence
	rs3783436	5		6 tissues	HRT,LIV		NF-Y	5 hits	5 hits	intronic	5 minimal binding evidence
SLC36A1 PAT1	rs357618	22					Egr-1		46 hits	intronic	6
	rs357629	22		FAT, SKIN	SKIN,SKN		Nkx3,Pou2f2	1 hits	46 hits	intronic	1f Likely to affect binding and linked to expression of a gene target
SLC38A2 SNAT2	*rs1873793*	9	HRT	17 tissues	10 tissues	4 bound proteins	6 altered motifs	1 hits	7 hits	intronic	2b likely to affect binding
	rs1858828	14	10 tissues	17 tissues	5 tissues		BDP1,Ets, RXRA		41 hits	291bp 5' of SLC38A3	4 Minimal binding evidence
SLC38A7 SNAT7	*rs9806843*	2					FAC1,NF-I	1 hits	3 hits	intronic	4 Minimal binding evidence

SLC38A9 SNAT9	*rs4865615*	324		FAT, MUS			4 altered motifs		3 hits	missense	6 Minimal binding evidence
	rs7704138	318						1 hits	4 hits	intronic	5 minimal binding evidence
	rs10056358	11			4 tissues		Egr1, SETDB1	2 hits		intronic	4 Minimal binding evidence

Abbreviations: Promoter/Enhancer histone marks, regulatory chromatin states based on ENCODE and Epigenomics Roadmap data; DNase, DNase hypersensitivity based on Epigenomics Roadmap data; Proteins bound, proteins bound by chromatin immunoprecipitation based on Epigenomics Roadmap data; Motifs changed, altered regulatory motifs; GRASP QTL hits, quantitative trait loci based on GRASP (Genome-Wide Repository of Associations Between SNPs and Phenotypes); selected eQTL hits, expression quantitative trait loci based on the Genotype-Tissue Expression (GTEx) analysis.

Scores indicate the following degrees of evidence: Score 1a, eQTL + TF binding + matched TF motif + matched DNase Footprint + DNase peak; Score 2b, TF binding + any motif + DNase Footprint + DNase peak; Score 3a, TF binding + any motif + DNase peak; Score 4, TF binding + DNase peak; Score 5, TF binding or DNase peak; Score 6, other; “No data” indicates that RegulomeDB holds no information about the given SNP, meaning there currently exists no evidence to suggest that the SNP has a regulatory function.
